# Unilateral Hypoglossal Nerve Palsy after Use of the Laryngeal Mask Airway Supreme

**DOI:** 10.1155/2014/369563

**Published:** 2014-08-31

**Authors:** Kenichi Takahoko, Hajime Iwasaki, Tomoki Sasakawa, Akihiro Suzuki, Hideki Matsumoto, Hiroshi Iwasaki

**Affiliations:** Department of Anesthesiology and Critical Care Medicine, Asahikawa Medical University, Midorigaoka Higashi 2-1-1-1, Asahikawa, Hokkaido 078-8510, Japan

## Abstract

*Purpose.* Hypoglossal nerve palsy after use of the laryngeal mask airway (LMA) is an exceptionally rare complication. We present the first case of unilateral hypoglossal nerve palsy after use of the LMA Supreme. *Clinical Features.* A healthy 67-year-old female was scheduled for a hallux valgus correction under general anesthesia combined with femoral and sciatic nerve blocks. A size 4 LMA Supreme was inserted successfully at the first attempt and the cuff was inflated with air at an intracuff pressure of 60 cmH_2_O using cuff pressure gauge. Anesthesia was maintained with oxygen, nitrous oxide (67%), and sevoflurane under spontaneous breathing. The surgery was uneventful and the duration of anesthesia was two hours. The LMA was removed as the patient woke and there were no immediate postoperative complications. The next morning, the patient complained of dysarthria and dysphasia. These symptoms were considered to be caused by the LMA compressing the nerve against the hyoid bone. Conservative treatment was chosen and the paralysis recovered completely after 5 months. *Conclusion.* Hypoglossal nerve injury may occur despite correct positioning of the LMA under the appropriate intracuff pressure. A follow-up period of at least 6 months should be taken into account for the recovery.

## 1. Introduction

Hypoglossal nerve injury associated with laryngeal mask airway (LMA) is a rare complication but can cause severe symptoms such as dysarthria and dysphasia. To our knowledge, eight cases of hypoglossal nerve palsy after use of the LMA have previously been reported [1–8]. In all these cases, LMAs which have silicone cuffs (e.g., LMA Classic and LMA ProSeal) were used. LMA Supreme (The Laryngeal Mask Company Ltd., St. Helier, Jersey, UK) is a disposable LMA which has anatomically curved airway and oval-shaped polyvinyl chloride (PVC) cuff. We present the first case of unilateral hypoglossal nerve palsy after use of the LMA Supreme.

## 2. Case Description

A 67-year-old female (weight 55 kg, height 155 cm) was scheduled for a hallux valgus correction. Her preoperative physical examination was normal and she had no past medical history (American Society of Anesthesiologists physical status I). No premedication was given. On arrival at the operating room, ultrasound-guided femoral and sciatic nerve blocks were performed under sedation with midazolam 2 mg and fentanyl 0.05 mg. After confirming the effects of blocks, general anesthesia was induced with propofol 3 mg*·*kg^−1^. A size 4 LMA Supreme was inserted successfully at the first attempt without difficulty using the standard insertion technique. The cuff was inflated with air at an intracuff pressure of 60 cmH_2_O using cuff pressure gauge (VBM Medizintechnik, Sulz, Germany). The LMA appeared to be in the correct position because there was gas leakage at a positive pressure of approximately 25 cmH_2_O and insertion of a gastric tube was smooth. Anesthesia was maintained with sevoflurane 1.5% and nitrous oxide 67% in oxygen under spontaneous breathing. The patient was in the supine position and hemodynamic parameters were stable during the surgery. The surgery was uneventful and the duration of anesthesia was two hours. The LMA was removed as the patient woke and there were no immediate postoperative complications.

**Figure 1:**
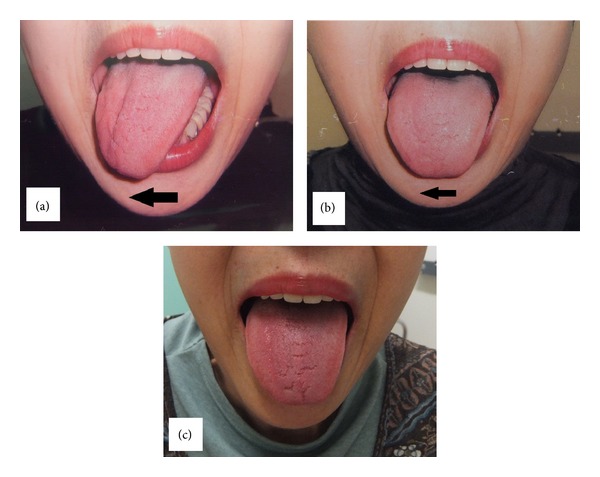
(a) Physical examination of the tongue on postoperative day 1. The tongue was deviated to the right side on protrusion demonstrating the right hypoglossal nerve palsy. (b) Physical examination of the tongue three months later. The deviation of the tongue slightly improved. (c) Physical examination of the tongue five months later. The deviation of the tongue disappeared showing a complete recovery of hypoglossal nerve function.

The next morning, the patient complained of difficulty in swallowing and slurred speech. Physical examination showed the deviation of the tongue to the right on protrusion (Figure 1(a)). The gag reflex and global and taste sensations of the tongue were normal. To exclude cerebrovascular diseases or internal carotid artery dissection, CT scan was performed, confirming the absence of abnormalities. These findings revealed isolated right hypoglossal nerve palsy after use of the LMA. In reference to previous reports [1–8], conservative management (including speech therapy and regular assessment) was chosen in the hope of spontaneous recovery. Medical follow-up was performed every 2–4 weeks. Although recovery was slow, the symptoms continued to improve and made a complete recovery after 5 months (Figures 1(b) and 1(c)).

## 3. Discussion

The LMA is one of the most widely used supraglottic airway devices. Although rare, neural damage such as lingual nerve injury [9], trauma to the recurrent laryngeal nerve [10], or hypoglossal nerve injury may be associated with an insertion of the LMA. On search of the literature, eight cases of hypoglossal nerve injuries associated with LMA have previously been reported [1–8]. One report was with LMA ProSeal [6] and all the others were with LMA Classic [1–5, 7, 8]. The LMA Supreme is the most recent type of laryngeal mask airway. This disposable device has a curved rigid stem copying the upper airway anatomy and allowing easy insertion and PVC cuff resistant to gas diffusion. As far as we know, neural damage associated with the LMA Supreme is extremely rare and only two cases of lingual nerve injury have been reported to date [11, 12]. We believe our report is the first case of unilateral hypoglossal nerve injury associated with LMA Supreme.

Potential risk factors for hypoglossal nerve injury are use of nitrous oxide, inappropriate size of the LMA, the lateral position, extreme head side rotation, primary illnesses, and difficult insertion of the LMA [13]. In our case, manufacturer's recommended size of the mask was chosen based on the body weight, the patient remained supine during the surgery and had no past medical history, and the mask was inserted successfully at the first attempt. Among the risk factors, only the use of nitrous oxide applied to our case. It is well known that nitrous oxide will diffuse into the LMA cuff and increase the cuff pressure with time during anesthesia [14]. The cuff pressure of LMA Classic and LMA ProSeal has been shown to increase from 60 cmH_2_O to over 100 cmH_2_O following 30 min of nitrous oxide exposure [14]. However, there are reports that LMAs with PVC cuff are less susceptible to hyperinflation caused by nitrous oxide compared to the ones with silicone cuff [15–17]. Anand et al. reported that intracuff pressure of LMA Supreme remained stable at approximately 60 cmH_2_O during an hour of nitrous oxide anesthesia [17]. Moreover, van Zundert et al. suggested that continuous cuff monitoring can be omitted in pediatric LMA with a PVC cuff during nitrous oxide anesthesia [18]. Therefore, excessive cuff pressure due to nitrous oxide anesthesia might not be the main factor which caused the nerve injury in our case.

Our case suggests that a correctly positioned LMA Supreme can occasionally cause a nerve injury. Correct position of the LMA was confirmed by the oropharyngeal leak pressure of approximately 25 cmH_2_O and easy gastric tube insertion [19]. Oropharyngeal leak pressure is the pressure at which a gas leak occurs around the LMA cuff. Oropharyngeal leak pressure in LMA Supreme at intracuff pressure of 60 cmH_2_O is reported to be approximately 20 cmH_2_O, similar to that in our case [20]. By any chance, rigid airway tube might have caused adverse effect. We postulate that the right hypoglossal nerve was compressed between the LMA cuff and the hyoid bone inadvertently during the anesthesia.

Hypoglossal nerve originates from the hypoglossal nerve nucleus in the medulla oblongata and leaves the skull through the hypoglossal canal of the occipital bone. It then descends between the internal carotid artery and the internal jugular vein. At the level of the angle of the mandible it becomes superficial, passes just above the greater horn of the hyoid bone, and enters the floor of the mouth [2]. Thus, a likely site of injury is at the greater horn of the hyoid bone where the inflated cuff of the LMA can compress the nerve against the bone [21]. The complete recovery of hypoglossal nerve injury has been reported to occur within the first 6 months, while there was one case of permanent recurrent nerve palsy after use of the LMA which resulted in no improvement [22]. These data suggest that the nerve function is temporarily impaired due to compression (i.e., neurapraxia). Depending on the extent of the exerted compression, the nerve may be permanently damaged due to collapse of its fibers (i.e., axonotmesis or neurotmesis). When the patient was diagnosed with hypoglossal nerve palsy caused by the LMA, a follow-up period of at least 6 months should be taken into account without invasive surgical procedures because spontaneous recovery is usually expected.
